# Analysis of sagittal plane cine magnetic resonance imaging for measurement of pancreatic tumor residual motion during breath hold and evaluation of gating margins used in radiotherapy treatment

**DOI:** 10.1002/acm2.14557

**Published:** 2024-11-04

**Authors:** Adam Phipps, Maxwell Robinson, Ben George, Tom Whyntie

**Affiliations:** ^1^ Department of Physics University of Oxford Oxford UK; ^2^ Department of Oncology, Old Road Campus Research Building University of Oxford Oxford UK; ^3^ Department of Radiotherapy Physics, Churchill Hospital Oxford University Hospitals NHS Trust Oxford UK; ^4^ GenesisCare Oxford Oxford UK; ^5^ Present address: Centre for Medical Imaging University College London London UK

**Keywords:** gating margins, MR‐guided radiotherapy, residual tumor motion

## Abstract

**Background and purpose:**

In pancreatic radiotherapy, residual tumor motion during treatment increases the risk of toxicity. Cine imaging acquired during magnetic resonance guided radiotherapy (MRgRT) enables real‐time treatment gating in response to anatomical motion, which can reduce this risk; however, treatment gating can negatively impact the efficiency of treatment. This study aimed to quantify the extent of residual tumor motion during breath hold and evaluate the appropriateness of the treatment gating margins used in current clinical practice.

**Materials and methods:**

Cine imaging acquired during pancreatic MRgRT of 11 patients on the ViewRay MRIdian was analyzed. The total duration of treatment analyzed was 12 h 13 min. Improved methods for processing and analyzing cine imaging were developed: breath holds were systematically separated with frequency analysis, residual motion was measured with consideration of both the tracking structure contour and centroid, and residual motion measurements were supported by phantom measurements of image scaling, resolution, and noise. Residual motion was measured at angles 0°, 45°, 90°, and 135° to the superior‐inferior (SI) direction. Total residual motion was measured by combining directional measurements.

**Results:**

The minimum tracking structure displacement resolvable through cine imaging was found to be 1.5 mm; therefore, residual motion analysis was limited to 1.5 mm spatial resolution. Total residual motion was contained within margins Δ=±1.5, ±3, and ±4.5mm with mean percentage frequencies of 97.0%, 91.1%, and 67.8%. Most residual motion was observed in the SI direction, and significantly more residual motion was measured for the tracking structure contour than the centroid.

**Conclusion:**

The results demonstrate that patients are largely able to maintain breath hold positions to within a 3 mm margin, thus provide evidence that supports the use of a 3mm gating margin in clinical practice. Residual motion frequently exceeded 1.5 mm so a reduction in gating margin would have an undesirable impact on treatment efficiency.

## 1 INTRODUCTION

The pancreas is subject to considerable respiratory motion and is situated amongst highly radiosensitive organs such as the stomach and duodenum; as such, accurate radiotherapy treatment is challenging and carries great risk of toxicity. To minimize toxicity, radiotherapy treatment is commonly delivered during breath hold, with each treatment fraction spanning many breath hold cycles. However, the stability and repeatability of tumor positions during breath hold is limited; variability in tumor position of ∼5mm has been measured in numerous previous studies with various measurement techniques.^1^, ^2^, ^3^, ^4^ Uncertainty in tumor position necessitates the use of motion encompassing treatment margins, which increases the risk of toxicity in neighboring organs.

Magnetic resonance guided radiotherapy (MRgRT) for pancreatic cancer offers many advantages over conventional treatment techniques.^5^, ^6^, ^7^, ^8^, ^9^ Continuous cine imaging acquired during MRgRT allows for real‐time anatomy tracking and treatment gating in which radiotherapy treatment is only administered the tracked anatomy returns to a specified position. This minimizes variability in tumor position during treatment and reduces the risk of toxicity. In clinical practice, a small tolerance (gating margin) in anatomical position is permitted before radiotherapy treatment is gated (switched off) as it is unachievable for patients to reproduce and maintain exact anatomical positions. The use of larger gating margins improves treatment efficiency as treatment can be delivered over a broader range of anatomical positions; however, larger gating margins increase the risk of toxicity in surrounding healthy tissue as they allow for more residual motion of the tumor during treatment delivery. Consequently, a compromise between treatment efficiency and the risk of toxicity is made when the gating margin is set. In MRgRT, patients can have a view of the real‐time cine imaging and anatomy tracking, which might reduce the extent of residual motion during breath hold compared to other respiratory‐gated treatments.

Previous studies have utilized cine‐MRI to measure the motion of abdominal tumors in both free breathing and breath hold conditions.^10^, ^11^, ^12^, ^13^ However, these studies used the centroid of the tracked tumor to measure tumor position, which potentially oversimplifies the motion of three‐dimensional tumors in soft tissue. Additionally, none of these studies considered the impact of anatomy tracking uncertainty on tumor position measurement or the limitations imposed by the finite resolution of cine imaging on the precision of their measurements.

This study aims to demonstrate improved methods for the analysis of cine imaging acquired during pancreatic MRgRT to quantify the extent of residual tumor motion during breath hold and evaluate the appropriateness of gating margins used in clinical practice. The methods presented in this study are supported by a series of phantom‐based experiments conducted to better understand the spatial resolution and uncertainty of cine imaging‐enabled anatomy tracking.

## 2 MATERIALS AND METHODS

### 2.1 In vivo data set

This retrospective analysis included all patients referred from a local institution for pancreatic MRgRT on the ViewRay MRIdian (ViewRay Inc. Cleveland, Ohio, USA). All patients were part of a service evaluation project approved by the local institution. Cine imaging acquired during treatment for 11 patients was analyzed in this study. The available data^14^ consisted of 55 cine imaging videos (4–7 treatment fractions per patient) with a combined duration of 12 h 13 min.

### 2.2 Cine imaging acquisition, anatomy tracking, and beam gating

Cine imaging on the MRIdian can be acquired in a single sagittal plane^15^ (Supporting Information Figure SF1). Table 1 outlines the frame rates and image geometries of the cine imaging included in this study; these imaging settings are all commonly used in pancreatic MRgRT at the local institution.

**TABLE 1:** Results from phantom experimentation on cine imaging.

						Tracking contour noise amplitude	
Image size (pixels × pixels)	Acquired in‐plane pixel spacing (mm × mm)	FOV (mm × mm)	Frame rate (fps)	In‐plane pixel spacing in exported cine imaging (mm × mm)	Minimum resolvable distance (mm)	$d_{95}^{AP}$(mm)	$d_{95}^{SI}$(mm)
144 × 144	2.4×2.4	345.6 × 345.6	8	0.806 ± 0.002	1.5	2.4	4.0
112 × 112	2.4×2.4	268.8 × 268.8	8	0.596 ± 0.002	1.25	2.8	5.4
100 × 100	3.5 × 3.5	350 × 350	4	0.792 ± 0.002	1.5	1.2	2.4

*Note*: Summary of results from phantom experimentation for each cine imaging setting. The measurement ‘minimum resolvable distance’ defines the minimum size of a tracking structure displacement resolvable through exported cine imaging. The quantities $d_{95}^{AP}$ and $d_{95}^{SI}$ refer to measures of tracking contour noise in the AP and SI directions respectively.

Abbreviations: AP, anterior‐posterior; SI, superior‐inferior.

The MRIdian implements real‐time anatomy tracking using deformable image registration (DIR). The tracking structure is defined as a sagittal plane cross‐section of the gross tumor volume (GTV) and is contoured on a high resolution three‐dimensional MRI scan prior to each treatment fraction. The initial tracking structure contour is expanded to form a larger gating boundary. A 3 mm gating margin is used as standard for pancreatic MRgRT on the MRIdian at the local institution since it aligns with GTV‐to‐PTV (planning target volume) expansion margin commonly used in pancreatic MRgRT treatment planning.^5^, ^6^, ^16^, ^17^

For each cine imaging frame, DIR is applied over a local region surrounding the GTV to propagate the tracking structure contour into an updated position. The system estimates the tracking confidence and the percentage of the tracking structure falling outside of the gating boundary. The radiotherapy beam is gated (switched off) if either one of two conditions are met: greater than a pre‐defined percentage of the tracking structure moves outside of the gating contour; tracking confidence falls below a pre‐defined threshold.

### 2.3 Image processing

Cine imaging acquired during treatment of each patient was exported from the MRIdian system as an OGV video file with resolution 512 × 512; the settings used during image acquisition were included in the bordering text of each exported video. Each frame was saved as a PNG image with resolution 512 × 512.

For each frame, the tracking structure contour was extracted by imposing thresholds on the array entries of separated RGB colour channels and the tracking structure centroid coordinates were calculated. The tracking confidence, gating state, and beam state were read from each frame. The accuracy of contour extraction from each cine imaging frame was validated using two independent methods (Supporting Information S1); the first method analyzed the shape of the extracted tracking structure contour, and the second method monitored the relative positions of the tracking structure and gating boundary.

### 2.4 Phantom experimentation

A series of experiments were conducted using a MR‐compatible motion phantom (Modus Medical Devices, Ontario, Canada) to better understand the MRIdian's cine imaging and anatomy tracking functionalities.

#### 2.4.1 Cine imaging scaling

Cine imaging videos are exported from the MRIdian with image size 512 × 512, requiring the original cine‐MRI images to be resized. Additionally, the original cine‐MRI images are reframed to accommodate the bordering text included in the exported videos (Figure SF1). For accurate measurement of tracking structure displacements, it was necessary to know the size, in mm, of the resized pixels in the exported cine imaging videos.

#### 2.4.2 Minimum resolvable distance

The ‘minimum resolvable distance’ was defined to describe the minimum size of a tracking structure displacement resolvable through exported cine imaging. This was important to measure as it defines the minimum spatial uncertainty on each extracted tracking structure contour.

#### 2.4.3 Noise in anatomy tracking

Noise in the cine imaging acquisition process hinders the efficacy of DIR and leads to noise in the tracking structure contour with frequency comparable to the cine imaging frame rate. To identify significant motion of the tracking structure, an understanding of the magnitude of this noise was necessary.

#### 2.4.4 Phantom measurements

The cine imaging scaling, minimum resolvable distance, and tracking contour noise were measured by acquiring cine imaging of the motion phantom (Supporting Information Figure SF3). Detailed accounts of the methods used for each measurement are included in the supplementary material (Supporting Information S2). These measurements were used to calibrate and define the spatial resolution of the following analysis.

#### 2.4.5 Noise reduction techniques

Noise in the tracking structure contour could be reduced by averaging the tracking contour over multiple frames. The motion phantom was used to develop time‐averaging methods for reducing noise in the tracking structure contour and centroid (Supporting Information S.2.4).

### 2.5 Analysis of in vivo data

#### 2.5.1 Separation of breath holds

Periods of breath hold were separated using Fourier frequency analysis on the motion of the tracking structure. Respiratory motion is periodic and is dominant in the SI direction; thus, the SI component of the tracking structure centroid velocity, $v_{SI}\left(t\right)$, was used to characterize respiratory motion. A Fast Fourier transform was applied to $v_{SI}\left(t\right)$ and periods of breath hold were identified by the absence of characteristic respiratory frequencies (Supporting Information S.3).

#### 2.5.2 Noise reduction

The trace of the tracking structure centroid during separated periods of breath hold was denoised using a first order Savitzky‐Golay filter with a time‐averaging interval of 2.5 s (Supporting Information S.2.4). The set of tracking structure contours extracted from each period of breath hold were denoised by finding the median tracking structure contour from normalized heat maps of tracking structure position generated from each 2.5 s time‐averaging interval.

Phantom experimentation justified the use of a 2.5 s time‐averaging interval for reduction of tracking contour noise to within 1.5mm. However, localization of the tracking structure to within 1.5 mm is only valid if the significant motion of the tracking structure within each time‐averaging interval amounts to less than 1.5 mm. A time‐averaging interval of 2.5 s therefore places an upper bound of 0.6mms^−1^ on the speed at which the tracking structure can move during breath hold. Use of a first‐order Savitzky‐Golay filter with an interval length of 2.5 s produced visually accurate centroid traces (Supporting Information Figure SF2) so this was used to characterize the bulk speed of the tracking structure during breath hold across all available cine imaging videos.

#### 2.5.3 Residual motion measurement

For each in vivo cine imaging video, residual motion of the tracking structure during breath hold was measured by consideration of both the tracking structure centroid and contour.

**FIGURE 1:**
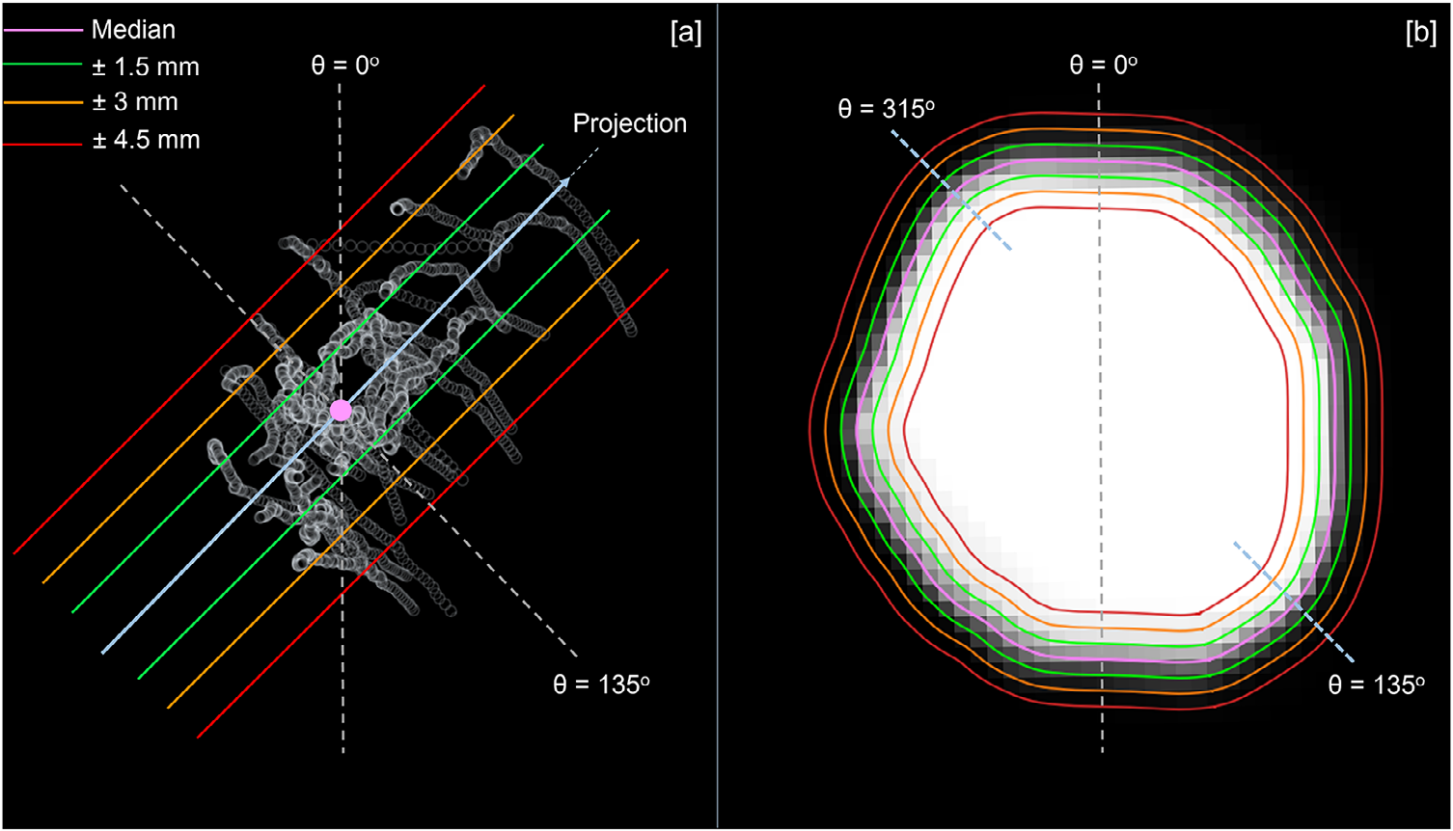
Measurement of tracking structure residual motion during breath hold at angle θ = 135° to the superior direction: (a) Projection of tracking structure centroid distribution, (b) Measurement of tracking structure heat map boundary width.

The distribution of denoised centroid coordinates was projected in multiple directions such that residual motion could be measured at angles 0°, 45°, 90° and 135° from the superior direction (Supporting Information Figure SF4). Projected centroid coordinates were grouped into spatial bins of width 1.5mm. The median centroid position defined the boundary between the two central‐most bins. The percentage of centroid coordinates contained within Δ=±1.5, ±3 and ±4.5 mm of the median position was calculated from the bin occupancies (Figure 1a).

A heat map of tracking structure position during breath hold with resolution 1.5 × 1.5 mm^2^ was created using the set of denoised tracking structure contours. Pixel values were normalized between 0–1 to represent the fraction of frames in which each pixel was contained within the tracking structure contour. The median position of the tracking structure contour was found by finding the contour at level 0.5 in the heat map. The percentage of denoised tracking structure contours falling within Δ=±1.5, ±3, and ±4.5 mm of the median tracking structure contour was then calculated for regions of the median tracking structure contour whose normals subtended angles of 0°, 45°, 90°, and 135° with the superior direction. For each angle, measurements were averaged with the corresponding antiparallel direction (Figure 1b).

The measurements for Δ≤3mm represent the fraction of time during breath hold that the tracking structure was contained within the 3mm gating boundary. To obtain an upper bound on this fraction, the minimum percentage for Δ≤3 mm across all angles was recorded. This was repeated for Δ≤1.5 mm and Δ≤4.5 mm to assess the implications of using 1.5 and 4.5 mm gating margins. The predicted influence on total treatment time was calculated from these results under the assumption that treatment time is inversely proportional to the frequency with which the tracking structure is contained within a given gating margin.

To analyze residual motion over the complete set of cine imaging videos available, a distribution of results was generated for each discussed measurement. Measurements were weighted by the total duration of breath hold from each video, and a weighted mean was calculated. A Wilcoxon signed‐rank test was performed for the residual motion measurement distributions at each Δ and measurement angle to compare the residual motion of the tracking structure contour and centroid.

## 3 RESULTS

### 3.1 Phantom experimentation

Table 1 summarizes the phantom experimentation results for each cine imaging setting relevant to the in vivo data set. The minimum resolvable distance was measured to the nearest 0.25 mm. These results show that sub‐voxel size displacements are resolvable using cine imaging. The quantities $d_{95}^{AP}$ and $d_{95}^{SI}$ refer to measures of tracking contour noise amplitude in the anterior‐posterior (AP) and SI directions, respectively. Considerable noise was measured, emphasizing the importance of noise reduction techniques for accurate residual motion measurement.

A time‐averaging interval of 2.5 s was sufficient to localize both the tracking structure contour and centroid to within a spatial margin of 1.5 mm (Supporting Information S.2.4). The minimum resolvable distance in the presence of tracking noise was taken to be 1.5 mm and defined the spatial resolution of the following residual motion measurements.

### 3.2 Analysis of in vivo data

The total duration of separated periods of breath hold from the available in vivo cine imaging data was 6 h and 18 min.

**FIGURE 2:**
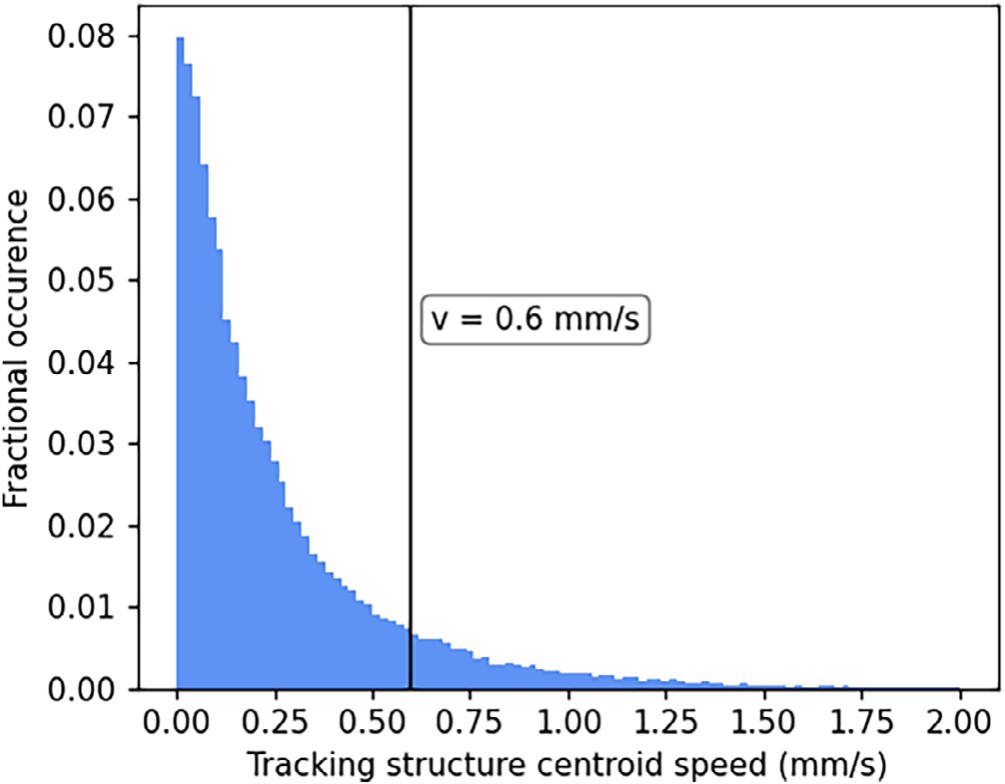
Distribution of tracking structure centroid speeds during all separated periods of breath hold from the available cine imaging data.

Figure 2 shows the distribution of tracking structure centroid speeds across all periods of breath hold. The distribution has mean and 95th percentile of 0.26 and 0.86 mms^−1^ respectively. It was found that 89.1% of tracking structure speeds during breath hold were less than the threshold of 0.6mms^−1^ derived from phantom experimentation; therefore, use of a 2.5 s time‐averaging interval is largely appropriate for noise reduction but may cause a slight undermeasurement of residual motion.

**FIGURE 3:**
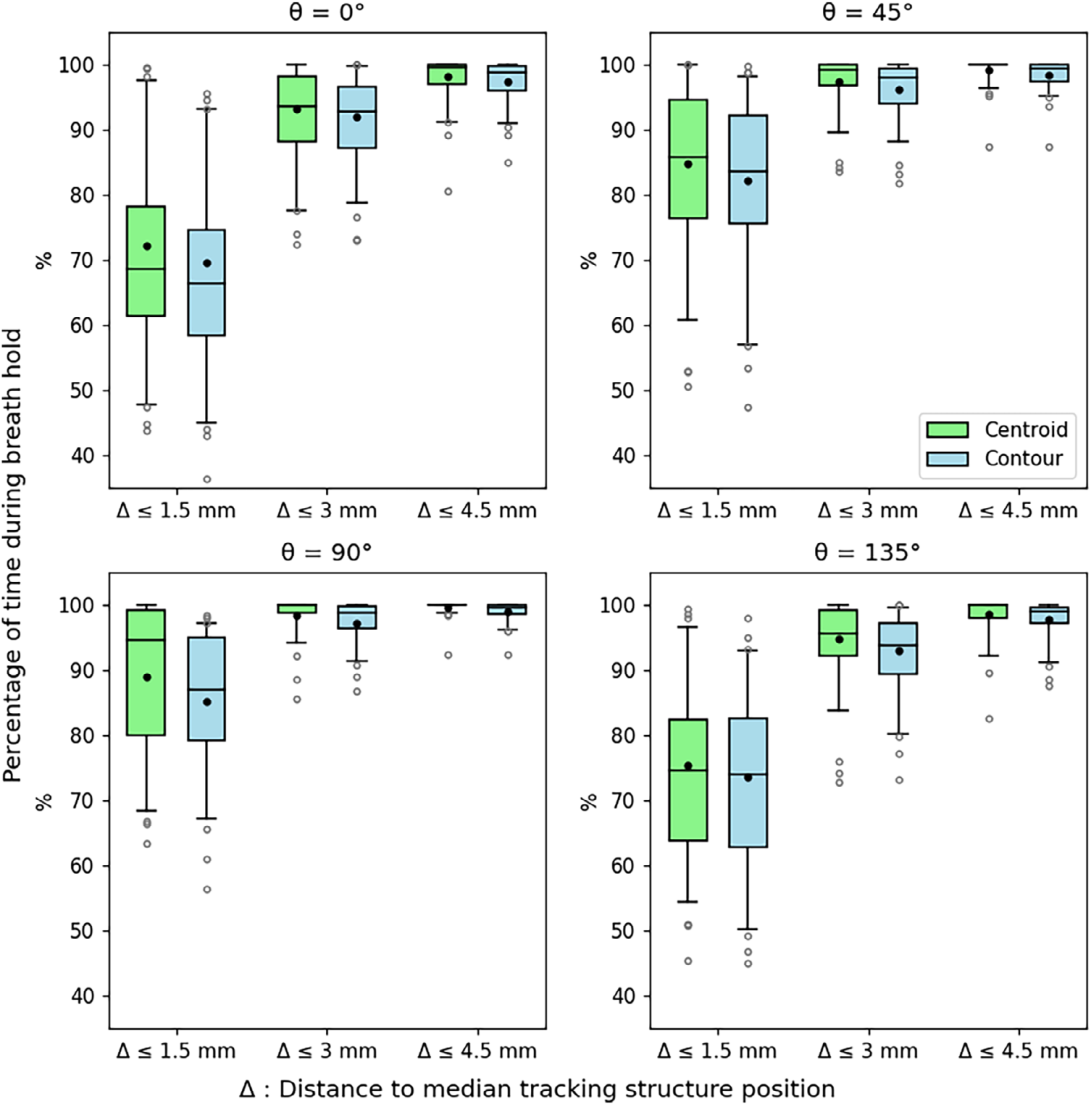
Residual motion of the tracking structure at angles θ = 0°, 45°, 90°, and 135° from the superior direction measured relative to the median tracking structure position across all separated periods of breath hold.

**FIGURE 4:**
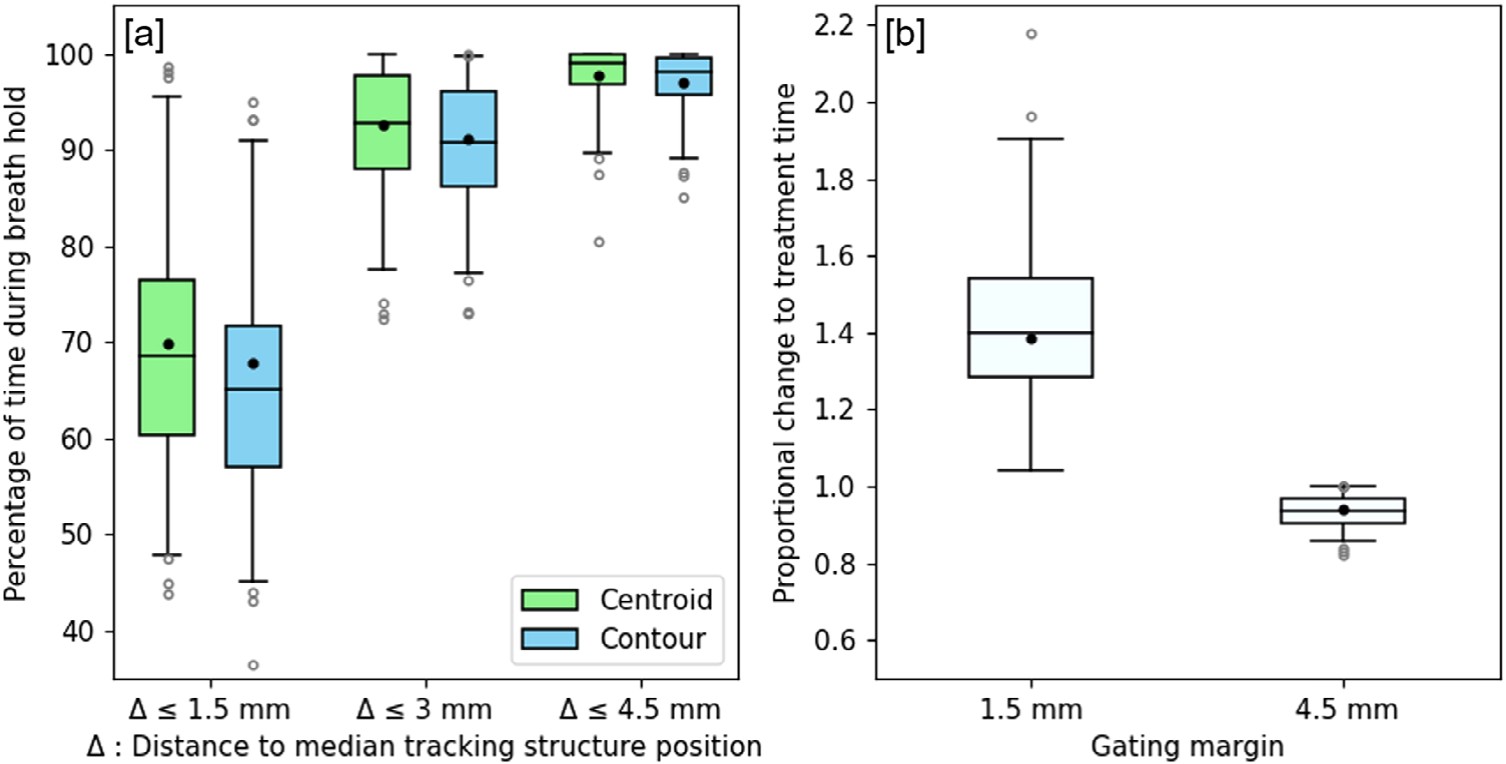
(a) Total residual motion of the tracking structure across all angles during separated periods of breath hold. (b) Predicted proportional change to treatment time with use of different gating margins.

Figures 3 and 4a show the results from directional and total residual motion measurements. Each boxplot displays the distribution of residual motion measurements across the in vivo data set with additional markings for the weighted mean of each measurement (weighted by breath hold duration). Overall, significantly more residual motion was observed for the tracking contour than the tracking structure centroid; statistical comparison of contour and centroid directional residual motion measurements yielded p<0.01 for all values of Δ and measurement angles θ. For total residual motion measurements at Δ= 1.5, 3, and 4.5 mm, *p* values were *p* = 2.3 × 10^−4^, *p* = 8.81 × 10^−5^, and *p* = 1.1 × 10^−4^, respectively.

The greatest residual motion of the tracking structure was observed at angles θ = 0° and θ = 135° to the superior direction, where the mean Δ≤3 mm frequencies for the tracking structure contour were 91.9% and 93.1%, respectively. In comparison, mean Δ≤3 mm frequencies at θ = 45° and θ = 90° were 96.1% and 97.3%. Mean frequencies for total residual motion of Δ≤1.5, 3, and 4.5 mm were 97.0%, 91.1%, and 67.8%.

Figure 4b displays the predicted influence on treatment time for use of 1.5 and 4.5 mm gating margins; a mean decrease of 6.2% is predicted for a 4.5 mm margin, whereas a mean increase of 38.5% is predicted for a 1.5 mm margin.

## 4 DISCUSSION

This study has presented improved methods for the measurement of residual tumor motion during pancreatic MRgRT. Frequency analysis of the tracking structure motion was used to automatically separate periods of breath hold; this proved to be an effective method since the amplitudes of characteristic respiratory frequencies were largely unaffected by frame‐to‐frame noise in the tracking structure contour. Phantom experimentation was conducted to calibrate and define the resolution of residual motion measurements; this ensured that measurements were accurate and were given to the appropriate precision. Phantom imaging was further used to investigate the impact of noise on residual motion measurements and to develop time‐averaging methods for noise reduction. This helped distinguish genuine motion of the tracking structure from noise, which ensured that the residual motion measurements recorded were a genuine reflection of tumor motion during breath hold. Phantom experimentation was important for meaningful analysis; however, the measurements of minimum resolvable distance and tracking noise illustrate the limitation of using cine imaging for precise measurement of tumor position. Residual motion was measured using both the centroid and contour of the tracking structure, which enabled an improved measurement of tumor motion to be obtained compared to previous studies; however, tumor motion orthogonal to the sagittal plane could not be directly measured using two‐dimensional cine imaging, so the presented residual motion measurements are still partially incomplete.

Significantly greater residual motion was observed for the tracking structure contour compared to the tracking structure centroid, which could be an indication of tumor motion orthogonal to the sagittal plane or variation in tumor shape. However, the techniques for denoising the tracking contour were developed using a phantom with sharp imaging boundaries, so the increased variation seen could also be attributed to an increase in noise while tracking structures with more ambiguous boundaries.

During phantom experimentation, the tracking structure was moved in the SI direction only; therefore, the performance of anatomy tracking in the AP direction was not measured directly. Despite this, we believe that the results from phantom experimentation remain informative for in vivo imaging since the majority of in vivo residual motion was seen in the SI direction.

Residual motion was found to be dominant at angles θ = 0° and θ = 135° to the superior direction. These are the dominant directions of respiratory motion; thus, residual motion can be attributed to inhalation depth variability between breath holds and diaphragm relaxation causing gradual retraction of breath hold positions.

 van Sörenson et al. measured 5th and 95th percentiles for residual centroid motion in the SI direction of ‐4.4 mm and 4.5 mm.^10^ In comparison, this study found that 98.1% of centroid positions were within 4.5mm of the mean centroid position, which suggests that less residual motion was observed for the patient cohort in this study. However, van Sörenson et al. included no consideration of anatomy tracking uncertainty or cine imaging resolution; therefore, tracking noise is a likely contributor to the greater residual motion seen and their presentation of residual motion measurements to 0.1mm precision is potentially misleading.

The total residual motion measurements show that patients were able to maintain >90% of breath hold positions to within a 3mm margin; these results support the use of a 3 mm gating margin for pancreatic MRgRT. The results do not advocate the use of 1.5 or 4.5 mm gating margins. Patients were considerably less able to maintain breath hold positions to within a 1.5 mm margin; as such, a significant increase in treatment time was predicted for the use of a 1.5 mm gating margin. In contrast, a small decrease in treatment time was predicted for a 4.5 mm gating margin; however, a 4.5 mm gating margin would allow for more residual motion during treatment and would increase the risk of toxicity.

Future work will investigate retrospective dose calculations that incorporate the residual motion measurement methodology presented in this study. This will enable a better understanding of treatment delivery accuracy and will be informative for deciding the appropriate GTV‐to‐PTV treatment planning expansion margin for pancreatic MRgRT. Further, this could help to standardize a protocol for pancreatic MRgRT treatment planning and could help to reduce the current variability seen between institutions.^18^ An understanding of beam gating latency will be important for this work.^19^, ^20^, ^21^, ^22^

In this study, residual motion was measured exclusively using the ViewRay MRIdian; the precision of residual motion measurements was limited by, and specific to, the anatomy tracking performance of the MRIdian system and the cine imaging settings outlined in Table 1. Acquisition of cine imaging with different imaging settings or using an alternative MR Linac system, such as the Elekta Unity, may permit more precise measurement of residual motion; however, neither of these changes would impact the magnitude of the underlying residual motion being measured, so the results presented in this study remain generally informative for determining the appropriate gating margin for pancreatic MRgRT.

## 5 CONCLUSION

The results from this study demonstrate that patients are largely able to maintain breath hold positions to within a 3 mm margin; this finding supports the use of a 3 mm gating margin in routine clinical practice. Patients were considerably less able to maintain breath hold positions to within a 1.5 mm margin. This indicates that, despite being beneficial for treatment accuracy, a reduction in gating margin would lead to a significant increase in treatment time, so may not be clinically favorable.

## AUTHOR CONTRIBUTIONS

This work was primarily completed by Adam Phipps under the supervision of Dr Tom Whyntie. Dr Ben George provided support in understanding the technical details of treatment on the MRIdian system and supervised the motion phantom experiments. Maxwell Robison provided insight into the clinical applicability of this work.

## CONFLICT OF INTEREST STATEMENT

The authors declare no conflicts of interest.

## Supporting information



Supporting Information
