# Supplementary material for: Analysis of sagittal plane cine magnetic resonance imaging for measurement of pancreatic tumor residual motion during breath hold and evaluation of gating margins used in radiotherapy treatment
Source: J Appl Clin Med Phys. 2024 Nov 4;26(1):e14557. doi: 10.1002/acm2.14557 (PMC11712868; doi:10.1002/acm2.14557)

**Supplementary Material**

**S.1 Error detection in contour extraction process**

***Error detection method 1***

For each cine imaging frame, the metric *perimeter/*$\sqrt{area}$ was calculated from the tracking structure binary mask. The mean µ and standard deviation σ were calculated from the distribution of metric values across all cine imaging videos. An extracted contour was considered erroneous if its metric value fell outside of the range µ ± 3σ. This range proved effective at detecting errors, whilst allowing for some variation in tracking structure shape.

***Error detection method 2***

As a secondary validation method, the percentage of the tracking structure falling outside of the gating boundary was independently calculated for each frame and compared against the value reported by the system. Due to differences in pixel size between the internal representation and images exported by the system, a difference of > 10% was deemed appropriate for detecting errors in the tracking contour extraction process.

The extracted tracking structure contours identified as erroneous were omitted from further analysis.

***Results***

Across all extracted frames from in vivo cine imaging, the total error rate in the contour extraction process was 1.10%. The mean individual cine imaging video error rate was 1.22% with standard deviation 1.35%.

**S.2 Phantom Experimentation**

A series of experiments were conducted using a Modus Quasar MRI 4D motion phantom (Modus Medical Devices, Ontario, Canada) to better understand the MRIdian’s cine imaging and anatomy tracking functionalities.

The motion phantom consists of an acrylic cuboid imaging volume submerged within a larger water-filled chamber. The acrylic volume generates negligible NMR signal compared to the surrounding water; a sagittal plane image of the phantom appears as a well-defined dark central rectangle surrounded by a much brighter exterior (Figure SF.3) A drive unit can be connected to the phantom, allowing the imaging volume to be longitudinally displaced over a range of 30mm within the bore hole of the MRIdian. The motion of the phantom can be controlled with user-written waveforms of displacement as a function of time.

Simple treatment plans were delivered to the phantom with the rectangular sagittal plane cross-section of the acrylic volume set as the tracking structure. The resulting cine imaging videos were then exported to investigate how the programmed motion of the phantom translated into observed motion through the cine imaging. This was done for all cine imaging settings relevant to the in vivo data set (outlined in Table 1).

**S.2.1** **Cine imaging scaling**

***Motivation***

Cine imaging videos are exported from the MRIdian with image size 512 x 512, requiring the original cine-MRI images to be resized and reframed. For accurate measurement of tracking structure displacements, it was necessary to know the size, in mm, of the resized pixels in the exported cine imaging videos.

***Method***

The imaging volume of the motion phantom was linearly displaced over a 30mm range for a duration of 180s. For each frame, the SI coordinate of the tracking structure centroid was calculated, and a linear fit was applied to the resulting centroid trace. The gradient of this linear fit represents the speed of the imaging volume in units pixels per frame. The ratio of this gradient and the true speed of the phantom, divided by the cine imaging frame rate, gives the size, in mm, of a pixel in the exported cine imaging video. The uncertainty on this measurement was calculated using the standard error on the linear fit.

***Results***

Results for measurement of pixel size in the exported cine images are included in Table 1.

**S.2.2 Minimum resolvable distance**

***Motivation***

An interpolation between pixels in the original cine MRI images is done by the MRIdian system to display a higher resolution image upon which the tracking structure is contoured. The finite resolution of the original and displayed images limits the minimum size of tracking structure displacements which can be resolved through the exported cine imaging. The 'minimum resolvable distance' was defined to describe the minimum size of a tracking structure displacement resolvable using exported cine imaging. This defines the minimum spatial uncertainty on each tracking contour extracted from the exported cine imaging.

***Method***

The phantom was displaced in a series of steps. Each step had an amplitude of 0.25mm and a duration of 20s. There were 28 steps in total, covering a range of 7mm. The set of tracking structure contours extracted during each step were denoised by implementation of the time averaging methods described in S.2.4, with the step duration set as the time averaging interval. The resulting denoised contours were overlaid on the same image and their spatial distribution was analysed.

***Results***

The set of denoised tracking structure contours clearly formed equally spaced bunches. The number of contours per bunch and the mean separation between bunches were measured to determine the size of the minimum resolvable tracking structure displacement to the nearest 0.25mm. The results of these measurements are included in Table 1.

**S.2.3 Tracking contour noise**

***Motivation***

Noise in the cine imaging acquisition process hinders the efficacy of DIR and causes noise in the tracking structure contour. This frame-to-frame noise has a frequency comparable to the cine imaging frame rate. To identify significant motion of the tracking structure, an understanding of the amplitude of noise in the tracking contour was necessary.

***Method***

The phantom was held stationary and imaged for 180s. For each frame, the tracking structure contour was extracted. The set of tracking contours from all frames was used to generate a heat map of tracking structure position with image size 512 x 512. Pixel values in the heat map were normalised between 0 and 1 to represent the fraction of frames in which each pixel was contained within the tracking contour. Contours at levels 0.025 and 0.975 in the heat map were found. The distance between these contours defines the width of a spatial interval within which the tracking contour was located for 95% of frames, thus, is a measure of random variability in the tracking contour. This distance was measured to the nearest 0.1mm along all 4 edges of the rectangular tracking structure. The maximum widths in the x and y directions, $d_{95}^{AP}$ and $d_{95}^{SI}$ were then recorded.

***Results***

Results for measurement of $d_{95}^{AP}$ and $d_{95}^{SI}$ in tracking contour noise are included in Table 1.

**S.2.4 Time averaging methods for noise reduction**

***Motivation***

The amplitude of noise in the tracking structure contour can be greatly reduced by averaging the tracking contour over multiple frames, enabling more accurate measurement of residual motion. The motion phantom was used to develop and evaluate time averaging methods for reducing noise in the tracking structure contour.

***Method***

A first order Savitzky-Golay filter was applied on both the x and y (AP and SI) components of the calculated tracking structure centroid trace from the stationary phantom imaging. This filter smooths the centroid trace by fitting a first order polynomial (linear fit) to successive sub-sets of points. The fitting is done using the method of linear least squares and the filter conserves the original number of data points. The time averaging interval, equal to the number of points in each subset divided by the cine imaging frame rate, was varied from 0.5s upwards and the spatial spread of the resulting set of time averaged tracking structure centroid coordinates was measured.

A normalised, tracking confidence weighted heat map (pixel values between 0 and 1, as described in section S.2.3) was produced using the tracking structure contours extracted from each time averaging interval during the cine imaging. Frames with greater MRI noise had a lower tracking confidence, thus, weighting a frames contribution to the time interval heat map using the tracking confidence quoted by the MRIdian improved noise reduction. The weighting of each contour was calculated by inputting the tracking confidence into a sigmoid function, constructed such that tracking confidences of 75%, 85% and 95% carried weightings of 0.025, 0.5 and 0.975 respectively. The median tracking structure contour position from this time interval was calculated by finding the contour at level 0.5 within the heat map. This was done for successive time intervals and the spatial spread of the resulting set of median contours was measured as described in section S.2.3. The time interval length used for time averaging was varied from 0.5s upwards.

***Results***

The Savitzky-Golay filter proved particularly effective for noise reduction in the phantom imaging. For cine imaging with original image sizes 100 x 100 and 144 x 144, a time averaging interval length of 1s was sufficient to localise 95% of tracking structure centroid coordinates to within an area of size (minimum resolvable distance)^2^: 1.5 x 1.5 mm^2^ for these image sizes. More noise in the tracking structure contour was seen for cine imaging with original image size 112 x 112, and the minimum resolvable distance for this cine imaging was found to be slightly smaller, at 1.25mm. As such, an interval length of 3s was required to achieve the same result. However, an interval length of 2.5s was sufficient to localise 95% of tracking structure centroid coordinates to within an area of size 1.5 x 1.5 mm^2^.

For cine imaging with original image size 144 x 144, a time averaging interval length of 2s was required to reduce the 95% spatial spread of the set of median tracking structure contours to less than the minimum resolvable distance. Interval lengths of 2.5s and 3.5s were required to achieve this result for cine imaging with original image sizes 100 x 100 and 112 x 112 respectively. For cine imaging with original image size 112 x 112, an interval length of 2.5s was sufficient to reduce the 95% spatial spread of median tracking structure contours to less than 1.5mm. Thus, the effective minimum resolvable distance for this cine was taken to be 1.5mm in the presence of tracking noise, and the analysis of residual motion was limited to 1.5mm resolution for all cine imaging settings.

**S.3 Breath hold separation with Fourier analysis**

Periods of breath hold were separated by implementation of Fourier frequency analysis on the motion of the tracking structure. Respiratory motion is periodic, and is dominant in the SI direction, thus, the SI component of the tracking structure’s centroid velocity, $v_{SI}\left( t \right)$, was used to characterise respiratory motion. A Fast Fourier Transform was applied to $v_{SI}\left( t \right)$; periods of breath hold were identified by the absence of characteristic respiratory frequencies.

A Fast Fourier Transform (FFT) was applied to $v_{SI}\left( t \right)$ over a sliding time interval of length $\Delta t = 8s$. This time interval was slid across the full domain $v_{SI}\left( t \right)$in increments of $\delta t=1/\mathrm{FPS}$. For each time interval, the Fourier frequency decomposition of $v_{SI}\left( t \right)$ was analysed. Steady state breathing was identified in intervals of duration > 5s over which the amplitudes of characteristic respiratory frequencies were dominant. A characteristic respiratory frequency range was chosen as 0.1-0.8 Hz, and dominance was determined if the area under the normalised FFT within this frequency range was greater than 0.6. These parameter values were chosen as they most clearly separated periods of breath hold in a subset of cine imaging videos used for testing. All separated periods of breath hold were visually checked against the SI component of the tracking structure centroid trace and, in cases of ambiguity, against the original cine imaging video.

**Figures**

Figure SF.1

**Title:** Example sagittal plane cine imaging frame.


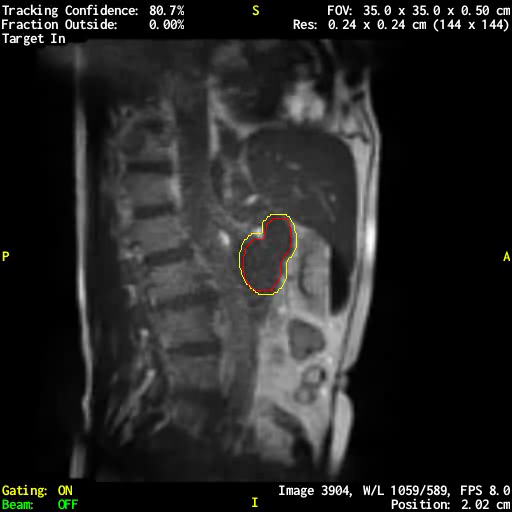


Figure SF.2

**Title:** Separation of breath holds using the SI component of the tracking structure centroid trace


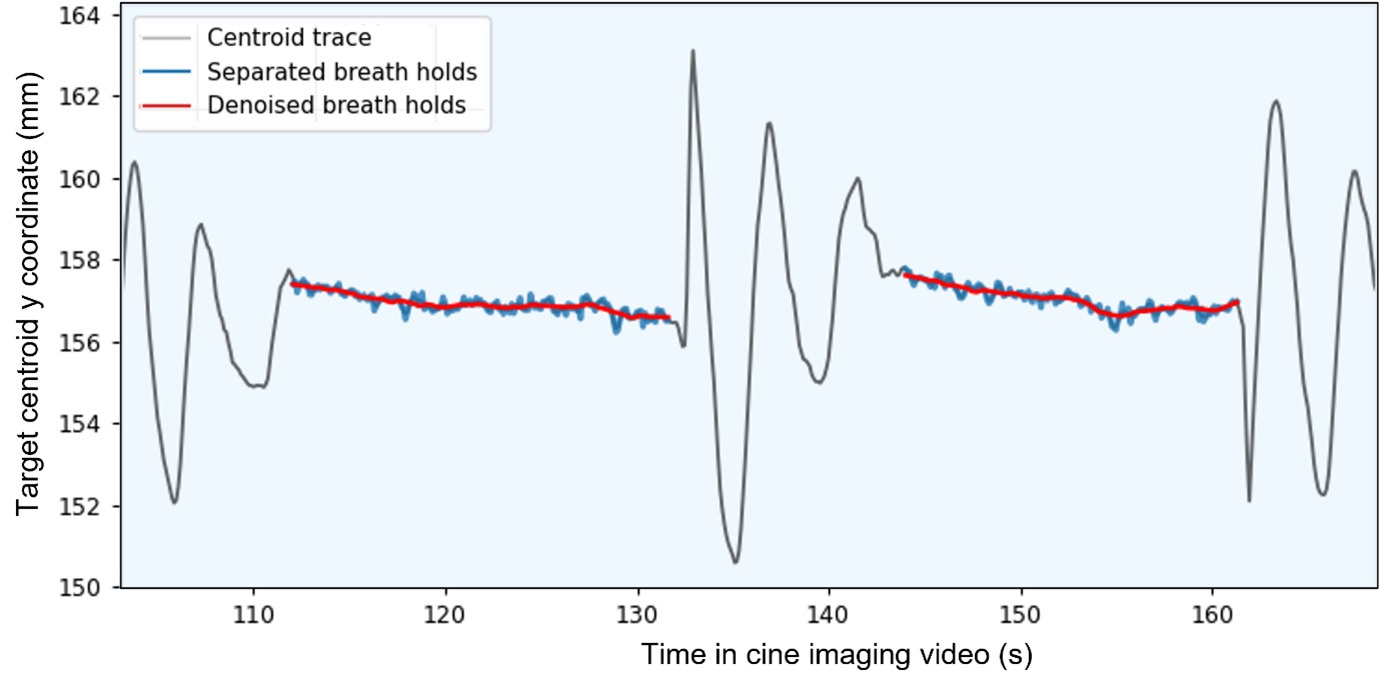


Figure SF.3

**Title:** Cine imaging of Modus Quasar motion phantom


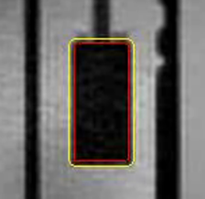


Figure SF.4

**Title:** Anatomical directions


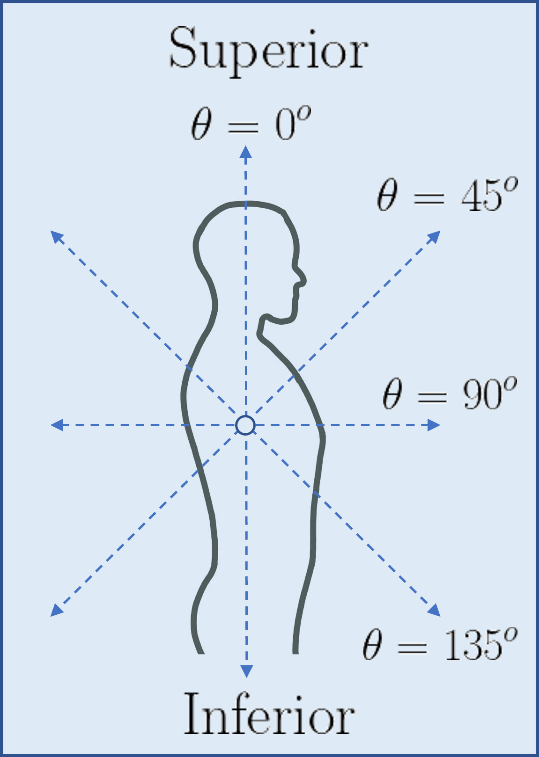

Supplement: Supplementary file 1 — Supporting Information [file ACM2-26-e14557-s001.docx]
